# Direct three-dimensional printed egg white hydrogel wound dressing promotes wound healing with hitching adipose stem cells

**DOI:** 10.3389/fbioe.2022.930551

**Published:** 2022-08-22

**Authors:** Xinhui Wang, Yuan Ma, Xingtang Niu, Ting Su, Xiaoqi Huang, Feng Lu, Qiang Chang

**Affiliations:** Department of Plastic and Cosmetic Surgery, Nanfang Hospital, Southern Medical University, Guangzhou, Guangdong, China

**Keywords:** wound healing, egg white derivative, 3D direct writing, adipose-derived stem cells, hydrogel

## Abstract

Current wound dressing based on hydrogel offers a promising way to accelerate the healing process, yet great challenges remain in the development of a highly integrated and efficient platform with the combination of therapeutic biomolecules and stem cells. Herein, a natural hydrogel wound dressing from egg white can be conveniently obtained by feasible physical crosslinking, the prepared hydrogel dressing features interconnected microporous channels, direct 3D printing, cytocompatibility, and intrinsic biomolecules to advance cell behavior. The 3D printed egg white hydrogels promote the adhesion and proliferation of adipose-derived stem cells (ASCs) without obvious cytotoxicity. In addition, this integrated hydrogel platform accompanied with adipose-derived stem cells accelerates wound healing through the enhancement of fibroblast proliferation, angiogenesis, and collagen rearrangement in the wound bed. The egg white hydrogel provides an effective wound caring product possessing low cost, easy availability along with ready manufacturing, and advanced therapeutic effect, which may be extended for the management of chronic or other complicated wounds.

## Introduction

Diverse biological cascades are activated when the skin integrity is disrupted, named hemostasis, inflammation, proliferation and remodeling (Sun et al., 2014). However, poor or delayed wound healing after extensive trauma or diabetes remains a major challenge, which prolongs pain and increases the risk of infection for the patient (Eming et al., 2017; Monika et al., 2022). More than 8.2 million people in the United States are eager for more advanced medical care for chronic wounds and subsequent comorbidities (Sen, 2019). Meanwhile, the chronic wound care market is expected to over 30 billion in the next 5 years because of population aging (desJardins-Park et al., 2021). The routine debridement supplemented with sterile gauze and negative pressure wound therapy can absorb exudate, but these techniques might leave the healing process on its own and have a limited benefit on the wound healing acceleration. In addition, frequent and incorrect manipulations may enlarge the original wound size and severity (Jones et al., 2018). The advanced and effective solutions to wound healing might relieve the lasting chronic wound and consequent sequelae for patients as well as the huge financial burden on public healthcare. Stem cell therapy has been evidenced as a promising option for wound healing promotion. Adipose-derived stem cells (ASCs) are renowned for easy access, abundance, long passage life and rapid proliferation compared with other sources (Hassanshahi et al., 2019; Zeng et al., 2022). After implantation into mammalian wounds, ASCs differentiate into repair-related cells ascribing to the pluripotent stemness. Moreover, a variety of functional factors (Such as growth factors, cytokines and exosomes) have been demonstrated by autocrine and paracrine of ASCs, which have exhibited a huge potential for immunoregulation, angiogenesis, fibroblast (Fb) proliferation and collagen remodeling (Li and Guo, 2018; An et al., 2021; Zhang et al., 2021; Zou et al., 2021).

The common strategies for ASCs delivery include intravenous injection, local intramuscular/intradermal injection or topical application (Zhou et al., 2021). However, direct contact with the wound environment or the shear force during injection might hinder the survival and the following function of stem cells. Meanwhile, functional molecules secreted from ASCs cannot be released or transported to the adjacent tissue stably and continuously, which might necessitate repeated treatment (Abdullahi et al., 2016; Han et al., 2022). As an alternative approach, encapsulation of ASCs into hydrogels and local application offers a considerable potential choice. Hydrogels are a stable three-dimensional network with high-water content, which provide a moist environment for wound healing. As the carrier of cell therapy, hydrogels protect the encapsulated cells from the shear force during injection and provide a favorable microenvironment for cell survival as well as achieve sustained release of cellular functional molecules (da Silva et al., 2019; Sivaraj et al., 2021). Notably, several obstacles remain to using hydrogels in clinical practice. For instance, the pore size of most hydrogels is non-porous or submicron, which might limit sufficient internal space to flow the oxygen and nutrients as well as excrete toxicants and metabolites for cells. Three-dimensional (3D) printing has occupied an important position as an advanced technology for macroporous hydrogels fabrication (De France et al., 2018). Unlike other bioprinting techniques, extrusion-based 3D printing can precisely control the pore size and overall structure of hydrogel scaffolds using a layer-by-layer deposition approach. Due to the existence of an ordered macroporous structure, 3D printing-based hydrogel showed better wound healing by improving the expression of ASCs behaviors in scaffolds (Wu Y. et al., 2021; Hu et al., 2021).

Ascribing to the low-cost and abundant resource, hydrogels derived from natural proteins and peptides have been extensively used from the bench to the bedside recently. Natural elements offer unique pharmacological performance to elevate the function of stem cells and accelerate wound healing through their bioactive factors (Bo et al., 2020; Wu T. et al., 2021; Liu et al., 2022). Egg white (EW) is an easily available natural nutriment that is essentially a diverse protein complex such as ovalbumin, ovotransferrin and lysozyme (Mann and Mann, 2011). Numerous studies have demonstrated the unique biological activities of proteins and peptides from EW, including anti-oxidative stress, anti-inflammatory activities and neovascularization (Zhou et al., 2022). Based on its protein nature, extensive research has been implemented to induce the EW liquid to form hydrogel and broadened its application spectrum.

**FIGURE 1:**
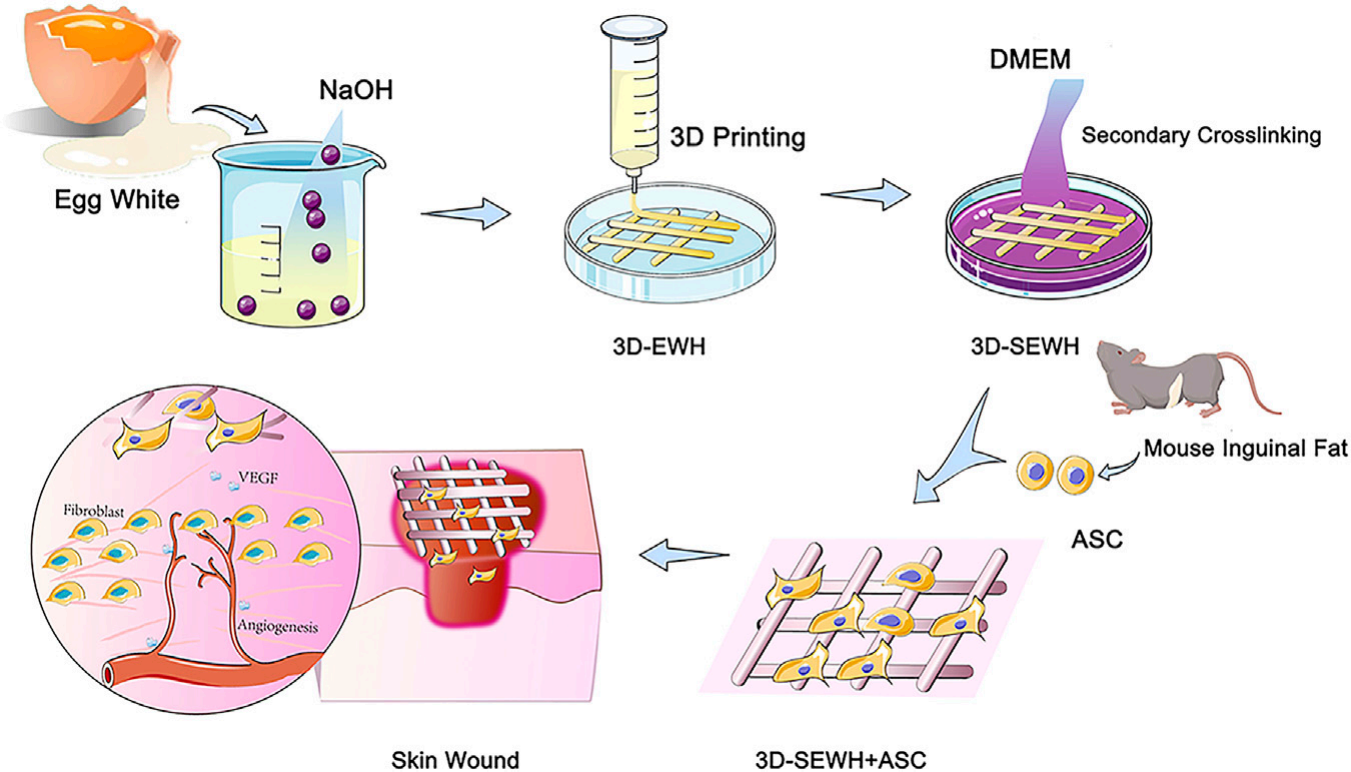
Schematic diagram of 3D-SEWH + ASCs for promoting wound healing.

In our previous study, we introduced a feasible method for the formation of physically crosslinked hydrogels based on EW in an alkaline environment (Chang et al., 2019). SEWH (EWH soaked in DMEM for secondary cross-linking) and 3D-SEWH utilized intrinsic bioactive components to stimulate the activity of Fbs and ASCs *in vitro*. Furthermore, 3D-SEWH showed better wound healing effect and angiogenesis than SEWH (Guo et al., 2022). However, the efficacy and clinical translation of EWH alone are still limited compared to relatively mature ASCs therapies. To further fulfill all the desired necessities of EWH wound dressing, herein, in this study we loaded 3D-SEWH with ASCs for better skin regeneration (Figure 1). ASCs in hydrogels exhibited higher viability, better functional factor secretion, and up-regulated Fbs migration and proliferation. After application to the skin wound healing mice models, ASCs and EWH synergistically modulate the inflammatory immune response, Fb behaviors, angiogenesis, and collagen remodeling. This study showed that 3D-printed EWH loaded with ASCs significantly accelerated wound healing.

## Materials and methods

### Materials and cell cultures

Cells used in this experiment include ASCs and L929 Fbs. ASCs were obtained from the inguinal fat of C57 mice, which were purchased from the Animal Center of Nanfang Hospital, Southern Medical University, Guangzhou, China. L929 Fbs was purchased from the Cell Bank of the Clinical Research Center of Nanfang Hospital, Southern Medical University, Guangzhou, China. Type 1 collagenase (Sigma-Aldrich, C0130), phosphate-buffered saline (PBS, Gibco™, 1X, pH 7.4), Red blood cell lysis buffer (Solarbio, R1010), etc. were used in the ASCs extraction process, and then ASCs were cultured in T25 cell culture flasks using mouse adipose-derived mesenchymal stem cell complete medium (Procell, CM-M138). L929 cells were grown in T25 cell culture flasks using high glucose Dulbecco’s modified Eagle’s Medium (DMEM, Gibco™,500 ml) supplemented with 10% fetal bovine serum (FBS, Solarbio, S9020) and 1% Pen/Strep solution (Gibco™ Penicillin-Streptomycin (10,000 U/ml), 15140122). All cell lines were kept in incubator at 5% CO_2_ pressure and 37°C (ESCO CelMate CO_2_ incubator). Hydrogels were prepared using sodium hydroxide (Aladdin, S111498) and DMEM (Gibco™,500 ml). For subsequent experiments, calcein (C110686) was purchased from Aladdin for cell live-death staining. 3,3’-dioctadecyloxacarbocyanine perchlorate (DIO) In biocompatibility experiments and CCK8 reagent in cell proliferation experiments were purchased from Beyotime. All cell experiments were performed in triplicate throughout the study.

### SEWH and 3D-SEWH fabrication and characterization

The preparation and characterization of egg white hydrogel were conducted according to protocols in our previous study with minor modifications (Chang et al., 2019; Guo et al., 2022). In previous studies, we demonstrated that the sodium hydroxide gel method has similar material and biological properties between different egg species (white-shell eggs, brown-shell eggs and duck eggs) (Guo et al., 2022). Considering the consistency and cost of this experiment, EW was extracted from fresh white-shell eggs and fully shaken evenly with a vortex vibrator to form a uniform EW liquid. Then, mixed it with sodium hydroxide (0.5 mol/L) in a ratio of 1:1 and stirred well to form a gel-like substance. The cross-linking mechanism was showed in Supplementary Figure S1. By using 3D printing technology (CREALITY 3D Ender-3, Shenzhen Creality 3D Technology Co., Ltd.) at room temperature (25°C), EWH was smoothly extruded from the needle tube at a speed of 1,400 mm/min under an air pressure of 75 psi and formed a standard mesh structure (2.5 cm x 2.5 cm x 0.2 cm) in the cell culture medium (Supplementary Video S1). At 4°C, soak in DMEM for 5°days (replace DMEM every 8 h) to neutralize sodium hydroxide and further enhance the structural stability of the hydrogel to form secondary crosslink egg white hydrogel. All steps are done on a clean bench to ensure the sterility of the hydrogel. SEWH loaded with ASCs was obtained by culturing mouse adipose stem cells with 1 × 10^5^ cells per gel on EWH soaked in a cell culture medium for 5°days. The following experiments were represented as the blank control group, ASC group (only ASCs), SEWH group (EWH + DMEM), 3D-SEWH group (3D-EWH + DMEM), and 3D-SEWH + ASC group.

To reveal the microstructure and spectra of EWH, EW, and SEWH from the same source were lyophilized for scanning electron microscope (SEM) and fourier transform infrared spectroscopy (FTIR) testing.

The gold sprayed SEWHs were observed with SEM (FEI, Nova NanoSEM 450, United States) at a working voltage of 15 kV.

FTIR spectra of two kinds of samples were recorded by the Nicolet 560 FTIR spectrometer equipped with Attenuated Total Reflection modular with the wavelength range from 4,000 to 400 cm^−1^ along with a 2 cm^−1^ resolution.

All rheological measurements were conducted on a TA Discovery HR-1 Rheometer with an 8-mm plate and with the proper gap between sample and substrate under room temperature and humidity. The storage and loss moduli were assessed by changing the strain from 0.1% to 500%, the viscosity was recorded as applying the shear rate between 0.1 and 100 s^−1^ for shear-thinning behavior.

### Biocompatibility of 3D-SEWH

Live-dead cell identification staining was used to detect the biological toxicity of SEWH to cells. 3D-SEWH was pre-incubated in the medium for 2 days (incubator, 37°C, 5% CO_2_) to obtain the supernatant. Then, the supernatant was added with 10% serum to prepare a mixed medium for co-culture with ASCs. In the control group, ASCs were cultured in an ordinary stem cell medium. The cells were stained with calcein working solution for 1 and 3 days, respectively. Washed with PBS before observed under an electric inverted fluorescence microscope (OLYMPUS IX73) and use cell counter (CounterStar; Rui Yu Biotechnology Co., Ltd., Shanghai, China) to analyze the percentage of cell viability. To evaluate the proliferation of ASCs in 3D-SEWH, add an appropriate volume of DIO to the ASCs and incubated at 37°C in the dark for 20 min. After staining, cells were added to culture dishes coated with 3D-SEWH and observed on day 1, 3, and 5 with a confocal microscope (Zeiss LSM980) and a motorized inverted fluorescence microscope (OLYMPUS IX73).

### Fibroblast proliferation

To investigate the effect of hydrogels on cell proliferation, we performed CCK8 experiments. 100 μl of mouse Fbs suspension with a density of 1 × 10^4^ cells/ml was prepared in a 96-well plate and pre-cultured for 24 h. Then, 100 μl of mixed medium containing 20 μl of supernatant from different groups incubated for 2 days was added to each well to replace the previous medium, and incubated in an incubator (37°C, 5% CO_2_). The absorbance at 450 nm was measured by a standard meter (BioTek ELX800 Microplate Reader) at 1–3 days.

### Mouse skin defect model

All animal experiments were approved by the Institutional Animal Care and Use Committee of Nanfang Hospital and were conducted in accordance with the guidelines of the National Health and Medical Research Committee (People’s Republic of China). And all applicable institutional and national guidelines for the care and use of animals were followed. 6-week-old male C57 mice were used as full-thickness excised skin defect models to investigate the potential of functional hydrogels on soft tissue wound healing. 60 mice were subjected to isoflurane-induced anesthesia. After removing the back hair, two full-thickness circular skin wounds with a diameter of 1 cm were excised on both sides of the back of each mouse. The wounds were covered with materials using different treatment methods (3D-SEWH, ASCs only, 3D-SEWH with ASCs), and then bandaged and fixed properly. Animals were then monitored daily, and the control group was also undergoing the same protection process to eliminate environmental differences. Animals were sacrificed on days 3, 5, 7, 11, and 14 after treatment, with three mice per group, and photographs of the wound site were taken, after which tissue samples were obtained and preserved in 4% PFA for histological studies. Hematoxylin and eosin (H&E) staining, Masson staining, and immunohistochemistry including VEGF and vimentin were used to explore angiogenesis and collagen deposition.

### Statistical analysis

All values were expressed as the mean ± standard deviation (SD). At least six independent experiments were carried out to assess reproducibility. Statistical significance was measured using a one-way analysis of variance (ANOVA) with Tukey’s posthoc analysis test. A *p* value <0.05 was considered statistically significant.

## Results and discussions

### Characterization of SEWH

**FIGURE 2:**
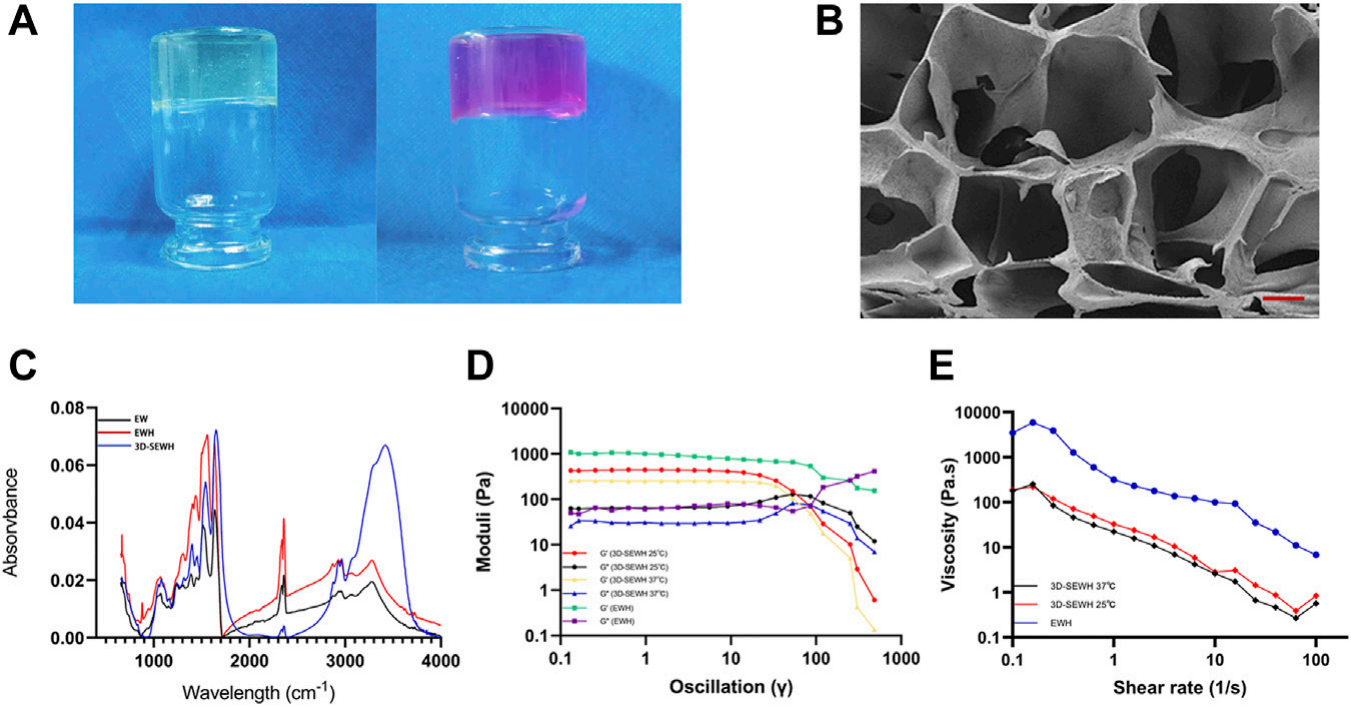
Material properties of SEWH. **(A)** Representative optical images before and after secondary crosslinking of EWH with DMEM. **(B)** Representative SEM images of the internal microstructure of SEWH, scale = 100 μm. **(C)** FTIR images associated with structural characteristics of EW, EWH and 3D-SEWH. **(D)** Rheological measurement of EWH and 3D-SEWH (25°C and 37°C). **(E)** Viscosity assessment of EWH and 3D-SEWH (25°C and 37°C).

The preparation of SEWH was composed of EW, sodium hydroxide (NaOH), and DMEM solutions. The EW for preparing hydrogels were derived from common white-shell eggs. Subsequently, EW and sodium hydroxide were mixed (concentration of 0.5 mol/L, mass ratio of 1:1) and quickly stirred to complete gelation. In order to improve the structural stability and biological properties, SEWH was prepared by soaking EWH in a DMEM solution for secondary crosslinking. Optical images showed that the appearance of EWH changed from white transparent to pink before and after DMEM crosslinking for 24 h (Figure 2A).

Pore structure is critical for the application of hydrogels in tissue engineering. SEM revealed the internal structure of SEWH, which formed a macroporous structure larger than 100 μm (Figure 2B). With large pore size and porosity, hydrogels are more conducive to the diffusion of soluble molecules (e.g., nutrients, waste and cytokines) and the expression of cell behavior (Annabi et al., 2010).

The spectral profiles of raw EW,EWH and 3D-SEWH were performed in the wavelength ranging from 4,000 to 400 cm^−1^ (Figure 2C). Similar peaks yet distinct feature could be found in these specimens, the broad peak around 3,200–3,300 cm^−1^ implied the O-H stretching at both samples. The absorption peaks of three major amide I, II, and III were located at the same area. In addition, the peaks of raw EW were located around 1,634, 1,522, and 1,238 cm^−1^, indicating the stretching vibration of C = O, stretching of N-H and vibration of C-N as well as C-N stretching, respectively. Meanwhile, after gelation with alkaline solution, the peaks of EWH changed a little left to higher wavelengths around 1,643, 1,559, and 1,250 cm^−1^, which was speculated a certain extent of disruption of original banding and rearrangement to form a new intermolecular hydrogen bonding during the gelation process and visualized as hydrogel formation. Similar to EWH, the peaks of 3D-SEWH were located around 1,652, 1,540, and 1,241 cm^−1^, and the broad peaks around 3,400–3,600 cm^−1^ were considered to be influenced by the -OH groups of phenols in DMEM.

The strain amplitude sweep tests was carried out to further assess the mechanical property of EWH and plotted (Figure 2D). The storage moduli (G′) and loss moduli (G″) were independent of the strain before the threshold where indicated the disruption of network under deformation. For EWH, G′ was higher than G″ (the values were around 1,020 Pa vs*.* 65 Pa) before the cross point (around 251%), suggesting the two moduli were kept at a certain constant level (in the linear viscoelastic region). As the strain increased, the sharp downturn of G′ and the uptrend of G″ met at the crossover point, which was the end of the linear viscoelastic interval. Then the gel structure changed and the viscoelastic network was demolished at the yield stress, resulting in a liquid-like behavior (Jiang et al., 2014). Therefore, this intersection could be considered as the yield stress, which was about 260 Pa. For 3D-SEWH, we performed the tests at 25°C and 37°C. Similar to EWH, before the intersection (about 53%, 25°C and 37°C), G′ was higher than G'' (values around 440 Pa vs. 63 Pa at 25°C; values around 250 Pa vs. 30 Pa at 37°C), which meant a gel-like or solid structure. The results showed that the gel networks of EWH and 3D-SEWH were damaged under different degrees of deformation due to their different structures. Meanwhile, the results also showed that there was no significant difference in the mechanical properties of 3D-SEWH at 25°C and 37°C.

The viscosity behavior at the function of shear rate of 3D-SEWH (25°C and 37°C) and EWH were shown in Figure 2E, the viscosity value of EWH decreased significantly with the shear rate increasing (ranging from 0.1 to 100 s^−1^), this shear-thinning performance might attribute to the physical and dynamic network in EWH and indicate this hydrogel could be injected under high shear rate and self-heal after the removal of shear force. While the viscosity curve pattern of 3D-SEWH was similar to that of EWH, the difference in value may be caused by the different physical structures of the two.

In rheological testing of hydrogels, we believe that the inevitable wall-slip phenomenon affects the measured values. It is worth noting that most of the published hydrogel articles to date default or ignore the effect of wall slip in rheological measurements, which obviously needed further elaboration. Kamkar et al. developed a technique chemically bonding and sandwiching two surfaces of a hydrogel to treated glass slides attached to the oscillating rheometer’s metal plates. Employing this method, they were able to completely alleviate errors attributed to the wall slip in the rheological measurements of soft materials (Kamkar et al., 2021, 2022). Therefore, in our study, we tried different methods to fix the 3D-SEWH on the plate of the rheometer to reduce the effects of wall slip. However, our current technical methods and present EWH cannot eliminate wall slip to achieve the most ideal detection conditions for the time being. This was a limitation for current study and we will conduct dedicated explorations in subsequent experiments to find more suitable methods to solve this problem.

### 3D printing and cytobiocompatibility of SEWH

**FIGURE 3:**
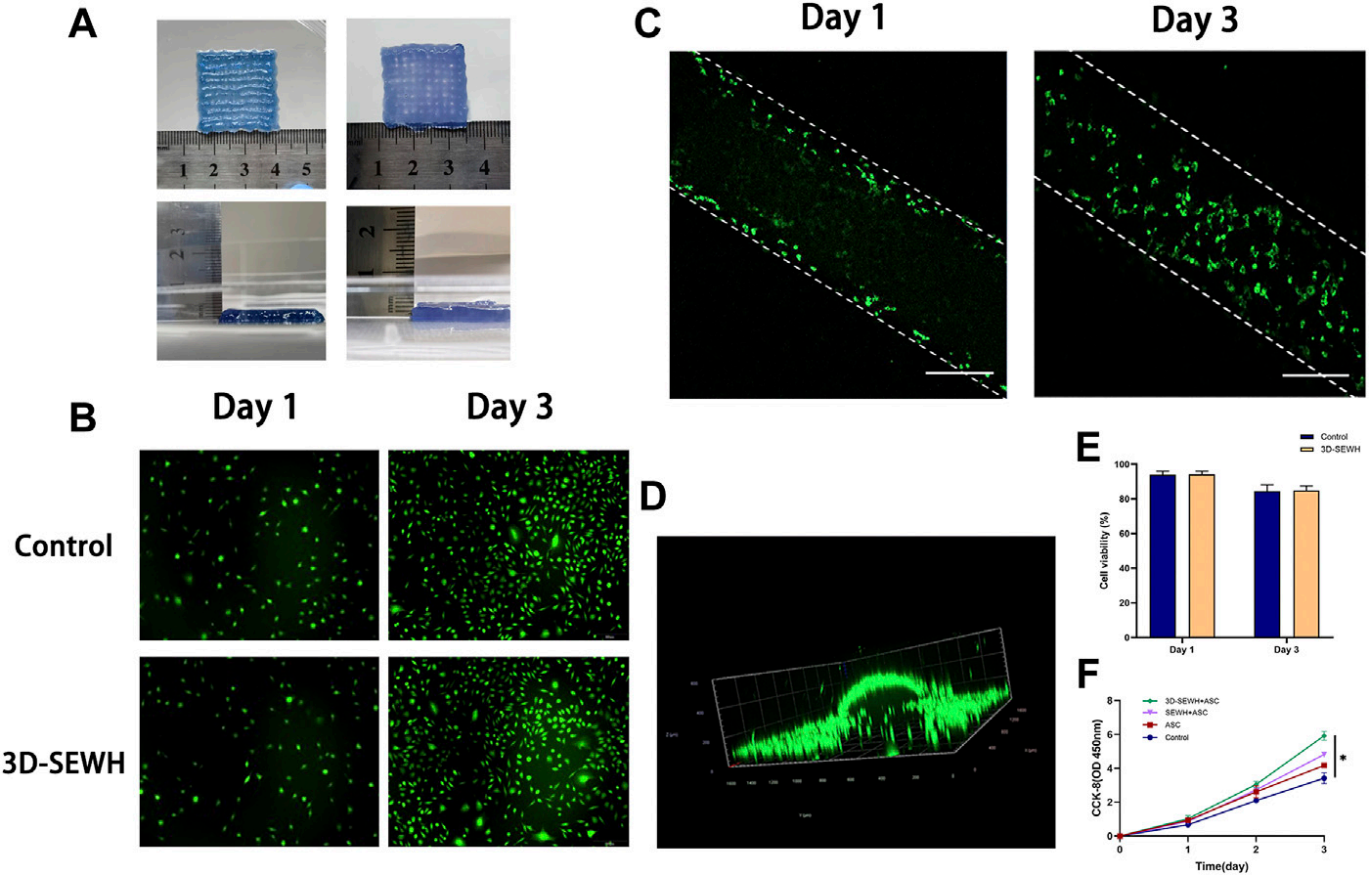
3D printing and cytocompatibility of SEWH. **(A)** Representative images of 3D-EWH and 3D-SEWH. **(B)** 3D-SEWH has no significant cytotoxicity to ASCs. **(C,D)** 3D-SEWH supports the adhesion and proliferation of ASCs, scale = 200 μm.**(E)** Cell viability percentage of 3D-SEWH and control group on day 1 and day 3. **(F)** Quantification of the effect of control, ASC, SEWH + ASC and 3DSEWH + ASC group on Fb proliferation, *p < 0.05.

Because of its superior accuracy and level of customization, 3D printing technology has been used to create wound dressings to provide better treatment for patients. The mesh formed by 3D printing provides the hydrogel with a pore structure that supports better cell and tissue growth. In addition, the grid-like topology functions to guide the directional migration of cells and tissues (Antezana et al., 2022). Based on the fast gelation property and shear thinning behavior, EWH was smoothly extruded from the needle tube and formed a standard mesh structure (2.5 cm x 2.5 cm x 0.2 cm) in the cell culture medium (Figure 3A). 3D-EWH was soaked in DMEM and changed every 8 h for 5 days to get a stable 3D-SEWH.

Good biocompatibility is an essential factor for the application of hydrogels. To explore the cytocompatibility of 3D-SEWH, supernatants were obtained from 3D-SEWH and added to cell culture dishes containing ASCs from C57 mouse fat tissues. Live-dead staining showed that a large number of green live cells were seen in both the control and 3D-SEWH group in day 1 with cell viability of 93% and 94.23%, respectively. Even on day 3, there were still a large number of viable ASCs in the 3D-SEWH group, and the cell viability was still 84.92%, which was not statistically different from the control group (Figures 3B,E). ASCs were seeded onto 3D-SEWH and co-cultured for 3 days. Confocal microscope images shown that ASCs were presented on the surface of the 3D-SEWH grid structure in day 1 and the number increased significantly in day 3 (Figure 3C). A 3D image further indicated that ASCs could stably adhere to the 3D-SEWH scaffold and proliferate normally (Figure 3D and Supplementary Video S2). Our results showed that 3D-SEWH had no obvious inhibitory or toxic effects on ASCs.

The enhancement of fibroblast function by ASCs has been widely reported. ASCs-derived extracellular vesicles have been shown to restore function in photodamaged skin by promoting fibroblast proliferation and collagen synthesis (Choi et al., 2019). ASCs also enhance fibroblast proliferation and migration through paracrine effects, which reduce cell apoptosis and senescence (Qin et al., 2020). As a carrier of stem cells, an ideal wound dressing can not only provide a homeostatic structure to maintain the physiological activities of cells, but also promote the function of cells through its own biophysical signals to accelerate wound healing. To evaluate the effect of 3D-SEWH + ASC on wound healing *in vitro*, we selected fibroblasts to examine the effects of proliferation. CCK-8 showed that fibroblasts stimulated by the supernatant of 3D-SEWH + ASCs possessed a higher proliferative rate than the control, ASCs and SEWH + ASCs groups (Figure 3F).

### 3D-SEWH + adipose-derived stem cells promoted full-thickness skin wound healing in mouse model

**FIGURE 4:**
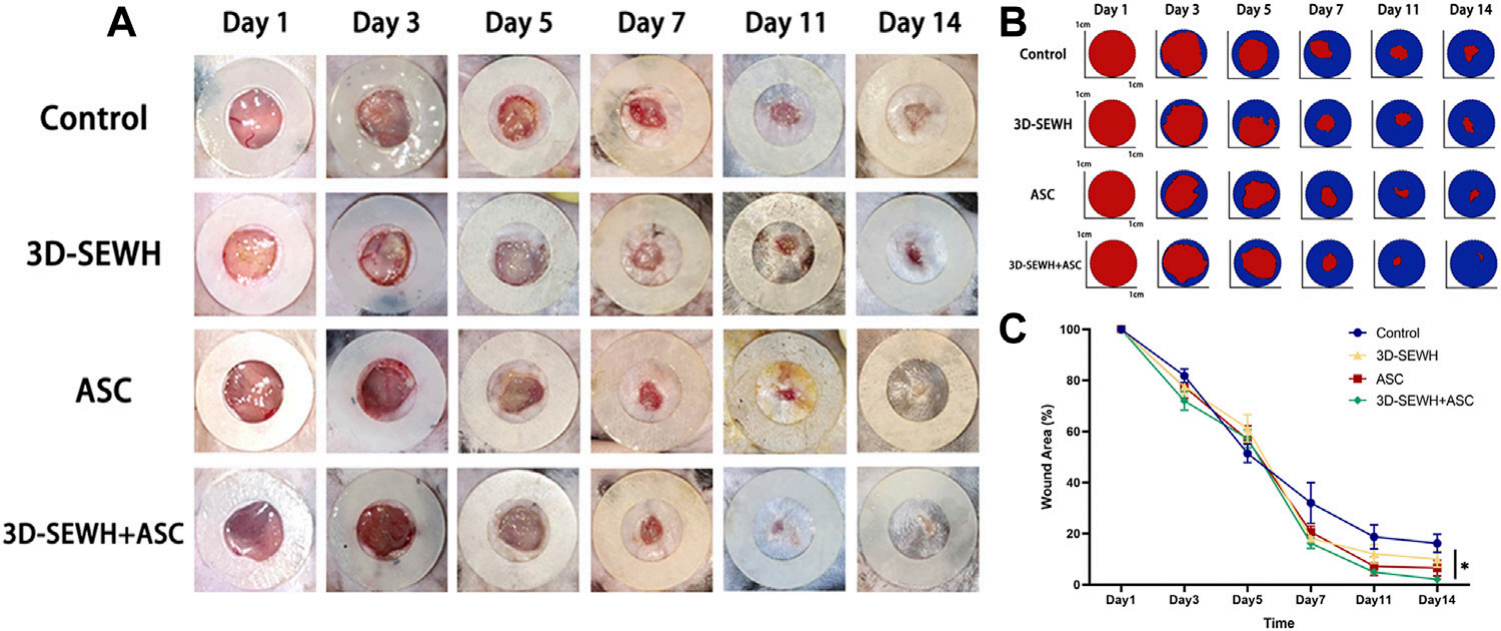
Therapeutic effect of 3D-SEWH + ASC in promoting full-thickness skin wound healing. **(A)** Representative images of skin regeneration. Mice wounds were divided into control, 3D-SEWH, ASC and 3D-SEWH + ASC according to different postoperative treatments. Images of wound areas were taken on days 1, 3, 5, 7, 11, and 14 and mice were sacrificed for section staining after taking pictures on day 14. The inner diameter of the annular silicone splint is 10 mm. **(B)** Skin regeneration pattern diagram. **(C)** Quantization of wound closure area, *p < 0.05.

3D printing is an emerging technology for the preparation of macroporous hydrogels, which can provide cells with precise adequate pore size and topology based on simple parameter tuning (De France et al., 2018). In addition, the 3D printed regular large pores can buffer swelling to reduce the risk of the dressing falling off the wound (Wu and Hong, 2019). Due to the advantages of 3D printing technology, we used 3D-SEWH in subsequent *in vivo* experiments for follow-up research work. The therapeutic effect of 3D-SEWH + ASC was further evaluated by the healing of the mouse full-thickness excision wound model. Healing trends were consistent among the four groups during the whole treatment. Representative optical and pattern images showed that the wound healing effect of 3D-SEWH was better than that of the control group after 14 days, but not as good as that of the 3D-SEWH + ASC group (Figures 4A,B). In the whole stage of wound healing, ASCs accelerated wound healing mainly in the remodeling stage rather than in the inflammatory and proliferative stages, which was based on the up-regulation of fibroplasia (Condé-Green et al., 2016; Franck et al., 2019). Our experimental results also support this view. In the first 5 days, the difference in wound healing area between the groups was minor. On the 7th day, compared with the control group (32.02% ± 7.98%), the 3D-SEWH group (20.64% ± 2.39%), the ASC group (18.12% ± 0.78%), and the 3D-SEWH + ASC group (16.21% ± 2.02%) had a significant decrease in wound area (Figure 4C). As the treatment progressed, the healing area of the 3D-SEWH group (12.04% ± 3.67%) and ASC group (7.22% ± 3.60%) decreased faster than the control group (18.72% ± 4.69%) in 11 days, but the effect was not as good as that of 3D-SEWH + ASC (4.90 ± 0.44%) group. On the 14th day, the 3D-EWH + ASC group almost completed the healing process, leaving only 2.10% ± 0.77% of the wound area. While the remaining unhealed wound area was significantly larger, which were 16.16% ± 3.60%, 10.02% ± 3.17% and 6.54% ± 3.09% in control group, 3D-EWH group and ASC group, respectively Although there was a time when the healing area of the control group was slightly lower than that of the other groups, the 3D-SEWH + ASC group had the best healing effect and time at 14s and 21 days.

### 3D-SEWH + adipose-derived stem cells promoted wound healing by regulating angiogenesis and collagen remodeling

**FIGURE 5:**
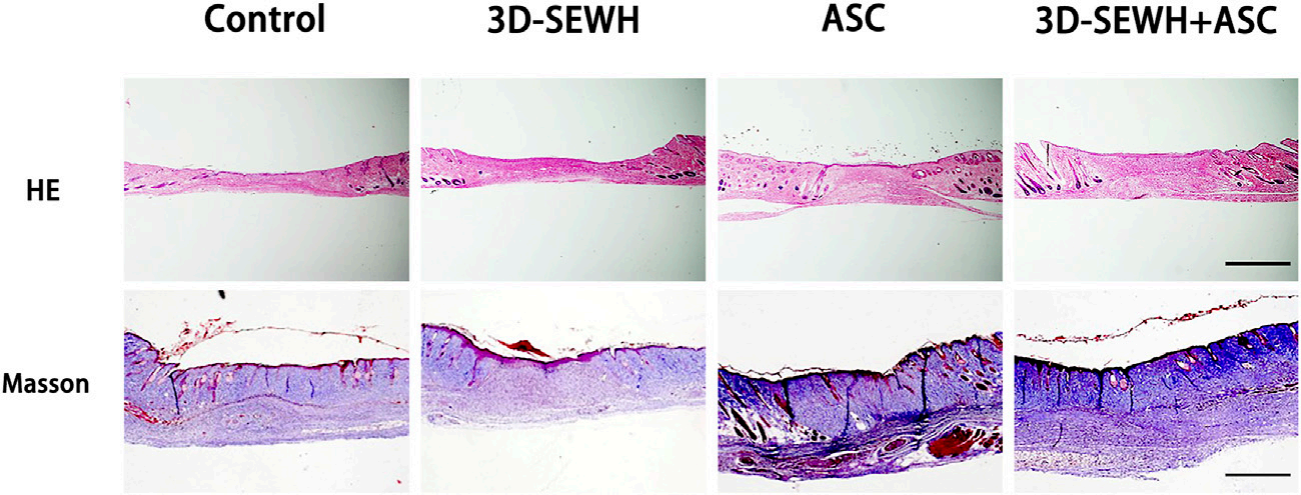
HE staining and MT staining of wounds on day 14 in each group. Scale = 500 μm.

The wound samples were collected from different groups on day 14 and stored in PFA for section observation. HE staining results showed that 3D-SEWH + ASC group had higher blood vessel distribution density and thicker new *epidermis* than other groups (Figure 5). Masson staining showed that the 3D-SEWH + ASC group had the most perfect collagen arrangement and the structure of each layer of the new *epidermis*.

**FIGURE 6:**
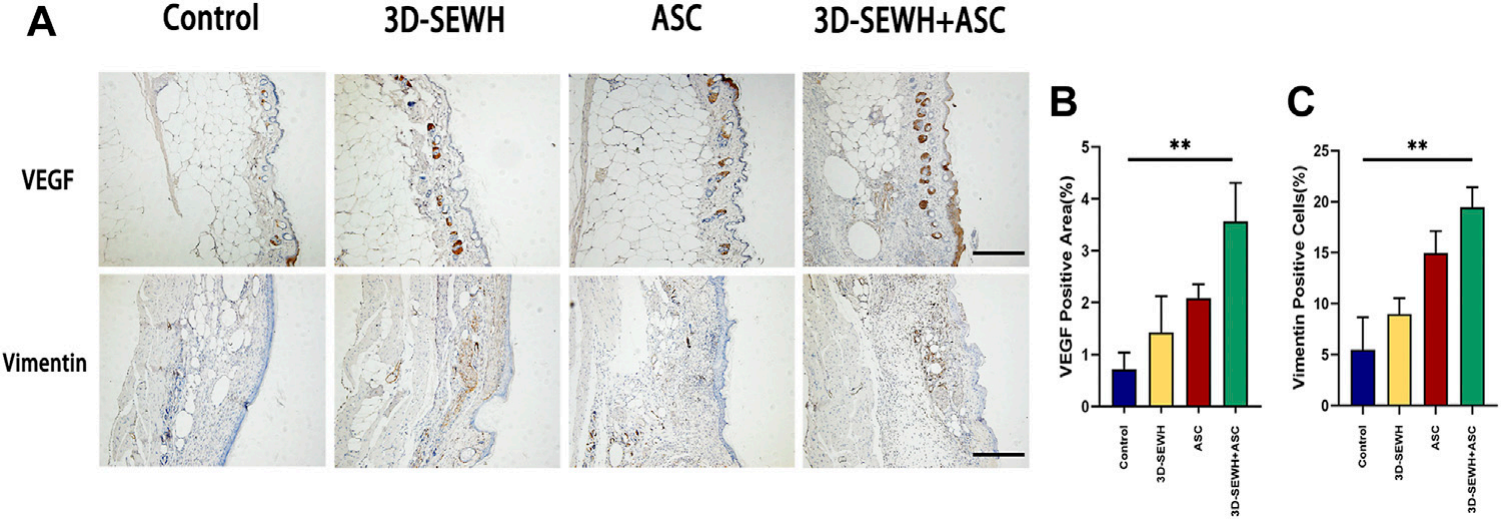
Potential mechanism of 3D-SEWH + ASC to improve healing efficiency in mouse wounds. **(A)** IHC staining of vascular remodeling and cell proliferation in different groups, scale = 200 μm. **(B)** Quantification of VEGF. **(C)** Quantification of Vimentin, **p < 0.01.

ASCs possess the ability to modulate inflammation and VEGF secretion. Furthermore, ASCs enhance vascular network remodeling by binding capillary and paracrine to recruit endothelial cells (Rockson, 2012). To further reveal the underlying mechanism by which 3D-SEWH + ASC promotes wound healing, we examined angiogenic factors and biomarkers of Fbs (Figure 6A). Immunohistochemical (IHC) results showed that the 3D-SEWH + ASC group had the highest number of positive angiogenesis and VEGF, which indicated that the enhancement of angiogenesis accelerated wound healing (Figures 6A,B). In addition, the number of vimentin-positive cells in 3D-SEWH + ASC was significantly increased, which indicated that the proliferation of Fbs in the wound was up-regulated and the dermal Fbs were more regularly arranged (Figures 6A,C). 3D-SEWH + ASC together improved wound repair and remodeling by modulating these underlying factors.

## Conclusion

In conclusion, we developed a 3D printed EWH as wound dressing to accelerate skin regeneration. The 3D-SEWH was obtained in the order of physical cross-linking of EW with NaOH, 3D direct writing and secondary cross-linking in DMEM, showing highly structural stability and advanced biocompatibility. The supernatant of 3D-SEWH and ASCs enhanced the proliferation of Fbs. The platform integrated 3D printed EWH and stem cells synergistically promoted angiogenesis and collagen rearrangement to facilitate wound healing. The proposed facile, economical yet highly effective platform from EW could be extended for more complicated tissue defects and might fuel the field of regenerative medicine forward from the bench to the bedside.

## Data Availability

The raw data supporting the conclusion of this article will be made available by the authors, without undue reservation.
